# Complexes of CO_2_ with the Azoles: Tetrel Bonds, Hydrogen Bonds and Other Secondary Interactions

**DOI:** 10.3390/molecules23040906

**Published:** 2018-04-14

**Authors:** Janet E. Del Bene, José Elguero, Ibon Alkorta

**Affiliations:** 1 Department of Chemistry, Youngstown State University, Youngstown, OH 44555, USA; 2 Instituto de Química Médica (IQM-CSIC), Juan de la Cierva, 3, E-28006 Madrid, Spain; iqmbe17@iqm.csic.es

**Keywords:** tetrel bond, hydrogen bond, carbon dioxide, azoles, IR spectra, spin-spin coupling constants

## Abstract

Ab initio MP2/aug’-cc-pVTZ calculations have been performed to investigate the complexes of CO_2_ with the azoles pyrrole, pyrazole, imidazole, 1,2,3- and 1,2,4-triazole, tetrazole and pentazole. Three types of complexes have been found on the CO_2_:azole potential surfaces. These include ten complexes stabilized by tetrel bonds that have the azole molecule in the symmetry plane of the complex; seven tetrel-bonded complexes in which the CO_2_ molecule is perpendicular to the symmetry plane; and four hydrogen-bonded complexes. Eight of the planar complexes are stabilized by Nx···C tetrel bonds and by a secondary interaction involving an adjacent Ny-H bond and an O atom of CO_2_. The seven perpendicular CO_2_:azole complexes form between CO_2_ and two adjacent N atoms of the ring, both of which are electron-pair donors. In three of the four hydrogen-bonded complexes, the proton-donor Nz-H bond of the ring is bonded to two C-H bonds, thereby precluding the planar and perpendicular complexes. The fourth hydrogen-bonded complex forms with the strongest acid pentazole. Binding energies, charge-transfer energies and changes in CO_2_ stretching and bending frequencies upon complex formation provide consistent descriptions of these complexes. Coupling constants across tetrel bonds are negligibly small, but ^2h^J(Ny-C) across Nz-H···C hydrogen bonds are larger and increase as the number of N atoms in the ring increases.

## 1. Introduction

Carbonic anhydrases belong to a family of enzymes that catalyze the reversible reaction that converts carbon dioxide and water to bicarbonate ion and a proton [1,2]. The proposed mechanism of action involves hydrogen-bond formation between the threonine N-H (Thr199) and a CO_2_ oxygen atom [3,4,5,6,7,8,9,10,11]. It is also known than azoles are good inhibitors of carbonic anhydrase [1,5,8,12,13,14]. Azoles are five-membered heteroaromatic compounds that contain in their rings from one nitrogen atom (pyrrole) to five nitrogen atoms (pentazole) [15]. The X-ray crystal structure of 1H-1,2,4-triazole bound through N2 to the N-H of Thr199 has been reported [16]. The interaction of CO_2_ with azoles is also important in carbon dioxide capture by metal-organic frameworks [17,18,19,20], zeolitic tetrazolate frameworks [21] and microporous organic polymers [22,23].

**Scheme 1:**
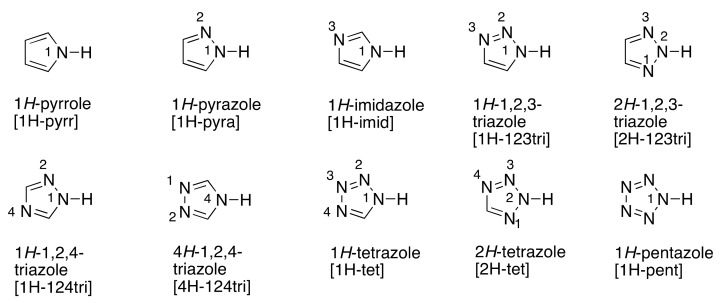
The ten azoles and their codes.

It is of interest to explore interactions that are important in biochemical reactions, such as those between azines and azoles with CO_2_. In a previous paper, we presented the structures, binding energies and other properties of CO_2_: azine complexes stabilized by N···C tetrel bonds [24]. The azoles have pyridine-like N atoms in positions 2, 3, 4 and 5 that may act as electron-pair donors as do the azines. However, the azoles also have pyrrole-like N atoms in positions 1, 2 and 4, as illustrated in Scheme 1. These N-H bonds may act as proton donors for hydrogen-bond formation.

**Scheme 2:**
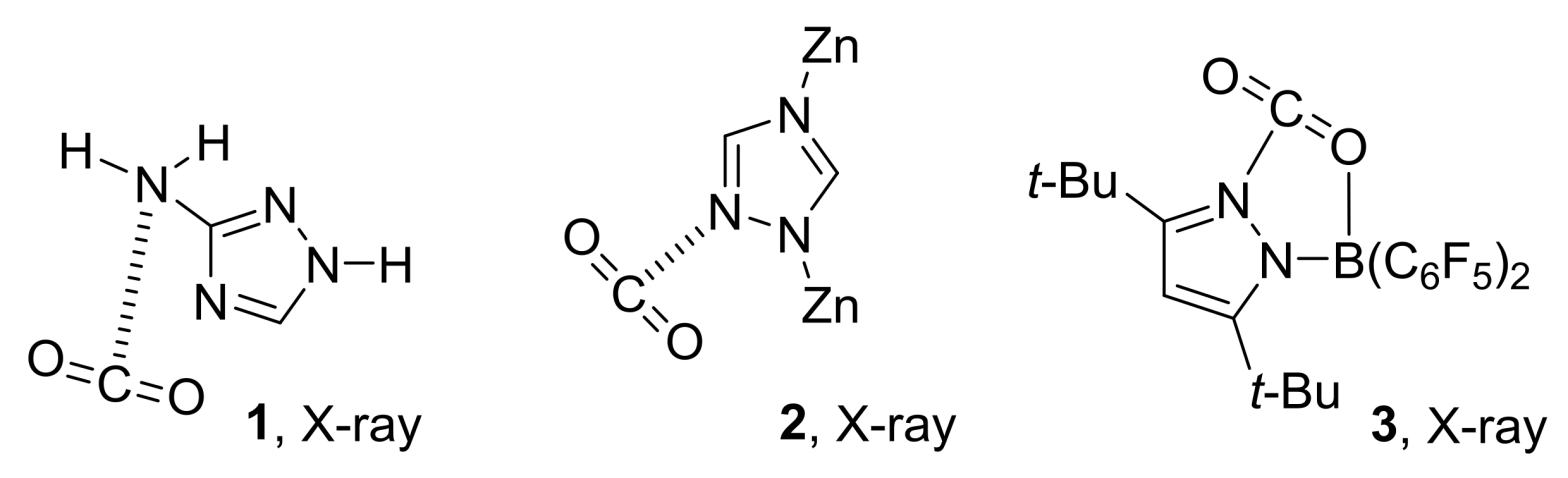
Relevant experimentally-determined complexes.

A series of papers have reported experimental data on complexes relevant to those investigated in the present study. The structures of these complexes are illustrated in Scheme 2. The X-ray crystal structure of the complex between 3-amino-1*H*-1,2,4-triazole and CO_2_ (**1**) shows a tetrel bond between the N of the amino group and the carbon of CO_2_ (CSD refcodes: WALBOC and YUZCED) [25,26]. A Zn-1,2,4-triazole derivative forms a tetrel-bonded complex with CO_2_ in the solid state using the free N2 atom of the triazole (**2**) (CSD refcodes: PEGBUA, PEGCAH, and PEGCEL) [27]. The hydrogen-bonded complex between a 2*H*-tetrazole and CO_2_ has been proposed to explain the behavior of a microporous organic polymer (CSD refcode: TZPIM) [22]. Finally, complex **3** is stabilized by an N-C covalent bond (CSD refcode: EPIVOQ) [28]. A related transition structure has been proposed that is stabilized by an N···C tetrel bond.

There are also three papers in the literature that report theoretical studies of complexes related to those investigated in this paper. Vogiatzis, Mavrandonakis, Klopper and Froudakis have reported MP2/aug-cc-pVTZ structures of complexes of CO_2_ with 1H-imid-23 and 2H-tet-34, with the CO_2_ molecule lying in the plane of the azole [29]. These authors noted the presence of stabilizing interactions involving an Hα atom of the azole and CO_2_. Prakash, Mathivon, Benoit, Chambaud and Hochlaf studied 1H-imid-23 and 1H-imid with CO_2_ and also investigated π stacking of imidazole [30]. Hernández-Marín and Lemus-Santana studied the complexes between CO_2_ and imidazole, 2-methylimidazole, benzimidazole and pyrazine using DFT methods [31]. Finally, Vidal-Vidal, Nieto Faza and Silva López extended studies of π-complexes to those with 1H-pyrrole, 1H-pyrazole, 1H-1,2,3-triazole, 2H-1,2,3-triazole, 4H-1,2,4-triazole, 2H-tetrazole and 1H-pentazole [32].

As a continuation of our work on intermolecular interactions, we present in this paper the results of a systematic study of complexes of the ten azoles with CO_2_. These include two types of tetrel-bonded complexes and a set of hydrogen-bonded complexes. We report the structures of these complexes, their binding energies and charge-transfer energies, selected IR stretching and bending frequencies and changes in these frequencies upon complexation, as well as spin-spin coupling constants across tetrel and hydrogen bonds. It is the purpose of this paper to present and discuss these results.

## 2. Methods

The structures of the isolated CO_2_ monomer, the azoles pyrrole, pyrazole, imidazole, 1,2,3- and 1,2,4-triazole, tetrazole and pentazole, and the complexes CO_2_:azole were optimized at second-order Møller–Plesset perturbation theory (MP2) [33,34,35,36] with the aug’-cc-pVTZ basis set [37]. This basis set was derived from the Dunning aug-cc-pVTZ basis set [38,39] by removing diffuse functions from H atoms. Frequencies were computed to establish that these optimized structures correspond to equilibrium structures on their potential surfaces and to examine the changes in selected vibrational frequencies upon complex formation. Optimization and frequency calculations were performed using the Gaussian 09 program [40]. The binding energies (−ΔE) of the complexes were computed as the negative of the reaction energy for the formation of the complex from CO_2_ and the corresponding azole.

The electron density properties at bond critical points (BCPs) of complexes have been analyzed using the atoms in molecules (AIM) methodology [41,42,43,44] employing the AIMAll [45] program. The topological analysis of the electron density produces the molecular graph of each complex that identifies the location of electron density features of interest, including the electron density (ρ) maxima associated with the various nuclei and saddle points that which correspond to BCPs. The zero gradient line that connects a BCP with two nuclei is the bond path. The natural bond orbital (NBO) method [46] has been employed to obtain the stabilizing charge-transfer interactions in complexes using the NBO-6 program [47]. Since MP2 orbitals are nonexistent, charge-transfer interactions have been computed using the B3LYP functional with the aug’-cc-pVTZ basis set at the MP2/aug’-cc-pVTZ complex geometries. This allows for the inclusion of at least some electron correlation effects.

Equation of motion coupled-cluster singles and doubles (EOM-CCSD) spin-spin coupling constants were evaluated in the CI (configuration interaction)-like approximation [48,49] with all electrons correlated. For these calculations, the Ahlrichs [50] qzp basis set was placed on ^13^C, ^15^N and ^17^O, and the qz2p basis set on the ^1^H atom bonded to N. The Dunning cc-pVDZ basis was used for the remaining H atoms. Total coupling constants were evaluated as the sum of the paramagnetic spin orbit (PSO), diamagnetic spin orbit (DSO), Fermi contact (FC) and spin dipole (SD) terms. Coupling constant calculations were performed using ACES II [51] on the HPC cluster Oakley at the Ohio Supercomputer Center.

## 3. Results and Discussion

### 3.1. Overview of the CO_2_:Azole Complexes

**Figure 1:**
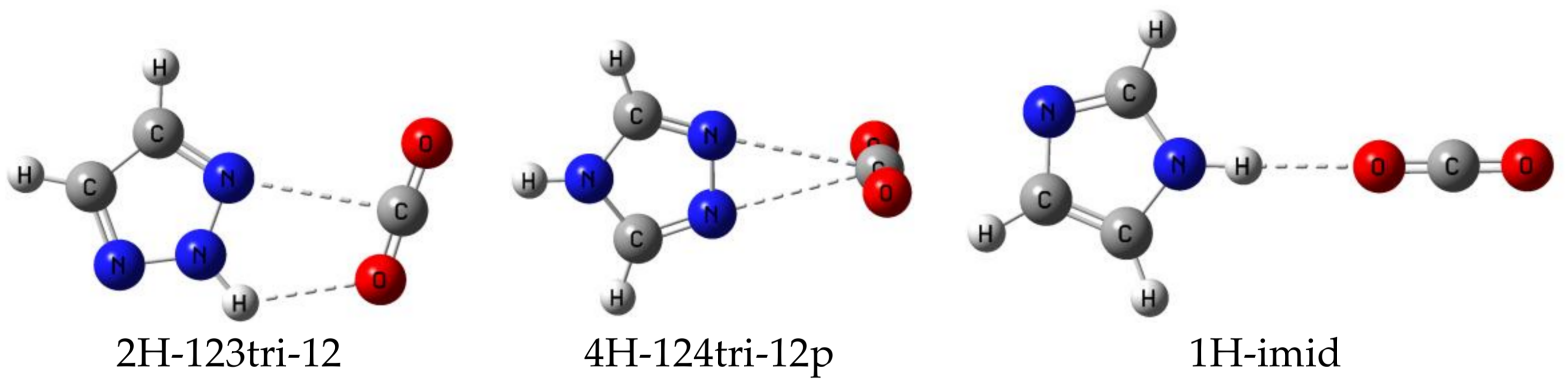
Representative tetrel- and hydrogen-bonded CO_2_:azole complexes.

Table 1 contains the names of the complexes, their binding energies and symmetries. The azoles are listed in Table 1 according to increasing number of nitrogen atoms. For each azole, the complexes are listed in order of decreasing binding energy. Three types of complexes have been found on the CO_2_:azole surfaces, namely tetrel-bonded complexes in which the CO_2_ molecule lies in the symmetry plane of the complex; tetrel-bonded complexes in which the CO_2_ molecule is perpendicular to the symmetry plane; and hydrogen-bonded complexes. Planar tetrel-bonded complexes are identified as zH-azole-xy, where z refers to the location of the Nz-H covalent bond in the ring, azole identifies the particular azole molecule and xy indicates the N atom that forms the tetrel bond and an adjacent N-H or C-H that may interact with CO_2_. The 2H-123tri-12 complex illustrated in Figure 1 is representative of planar tetrel-bonded complexes. For complexes in which the CO_2_ molecule is perpendicular to the symmetry plane, the designation is similar, with xy referring to adjacent N atoms that have lone pairs of electrons and p indicating a perpendicular complex. 4H-124tri-12p in which N1 and N2 donate lone pairs for tetrel-bond formation is typical of these complexes and is also illustrated in Figure 1. Complexes in the third set are not tetrel-bonded, but hydrogen bonded, and are identified as zH-azole. The N1-H···O hydrogen-bonded complex 1H-imid is illustrated in Figure 1.

### 3.2. Planar Complexes Stabilized by Tetrel Bonds

**Figure 2:**
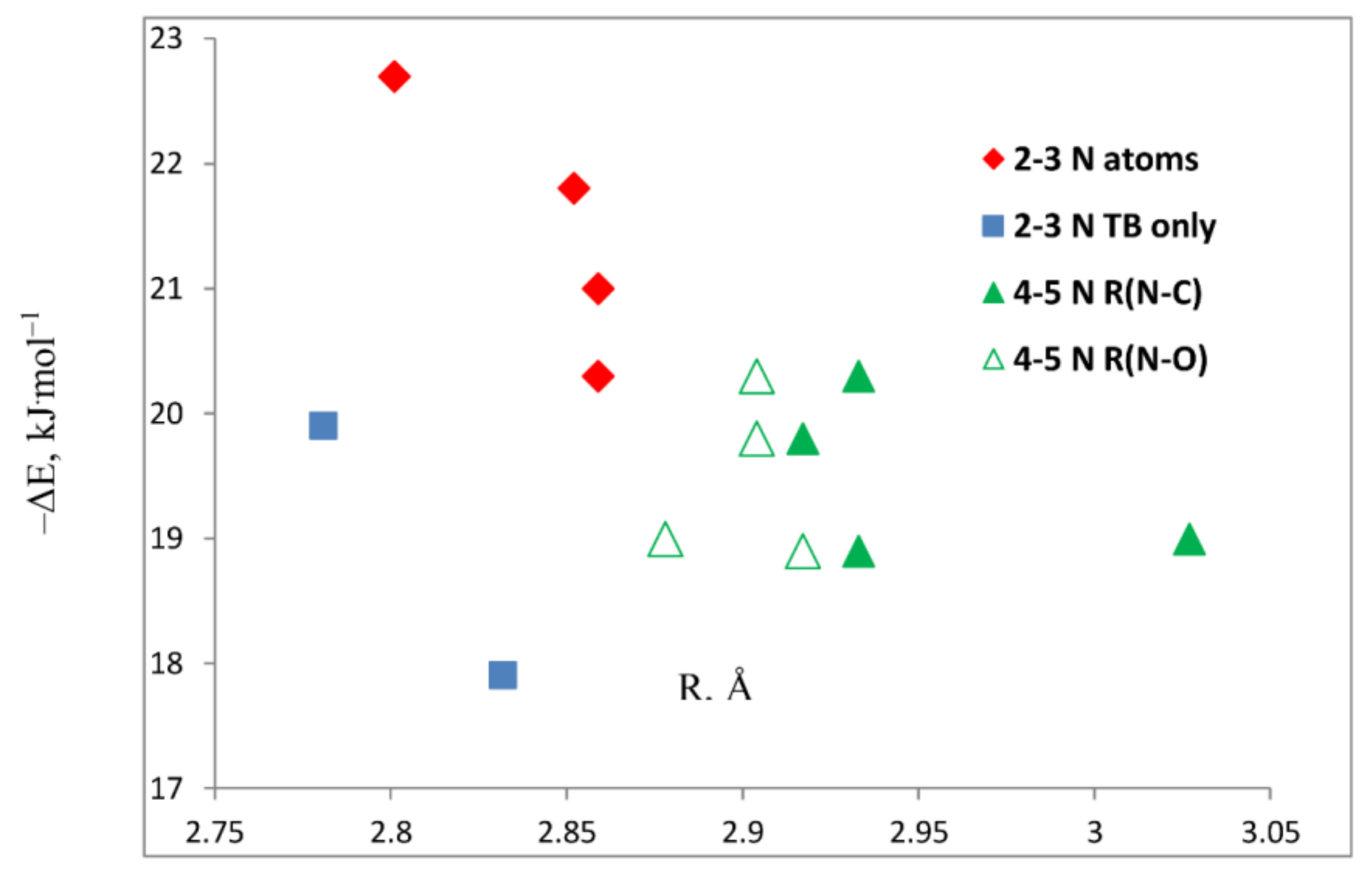
Binding energies versus distance for the planar tetrel-bonded CO_2_:azole complexes. The solid symbols refer to the Nx-C distance; open symbols refer to the Ny-O’ distance in complexes with tetrazole and pentazole.

The structures, total energies and molecular graphs of planar complexes stabilized by tetrel bonds are reported in Table S1 of the Supporting Information. Table 2 reports their binding energies, charge-transfer energies and Nx-C, Ny-O’ and O’-H, or Cy-O’ and O’-H distances, with O’ the adjacent atom of CO_2_. The binding energies of these complexes vary by less than 5 kJ·mol^−1^, from 17.9 kJ·mol^−1^ for 1H-124tri-45 to 22.7 kJ·mol^−1^ for 1H-pyra-12. The Nx-C distances range from 2.781 Å in 1H-imid-23 to 3.027 Å in 1H-pent-12. However, the binding energies do not correlate with the Nx-C distances, as can be seen from the scattergram of Figure 2. To gain insight into Figure 2, it is advantageous to subdivide the planar tetrel-bonded complexes into three groups: those in which Cy-H is adjacent to Nx; those in which Ny-H is adjacent to Nx and have an Nx-C distance that is shorter than the Ny-O’ distance; and those that also have Ny-H adjacent Nx, but have an Ny-O’ distance that is shorter than the Nx-C distance.

**Figure 3:**
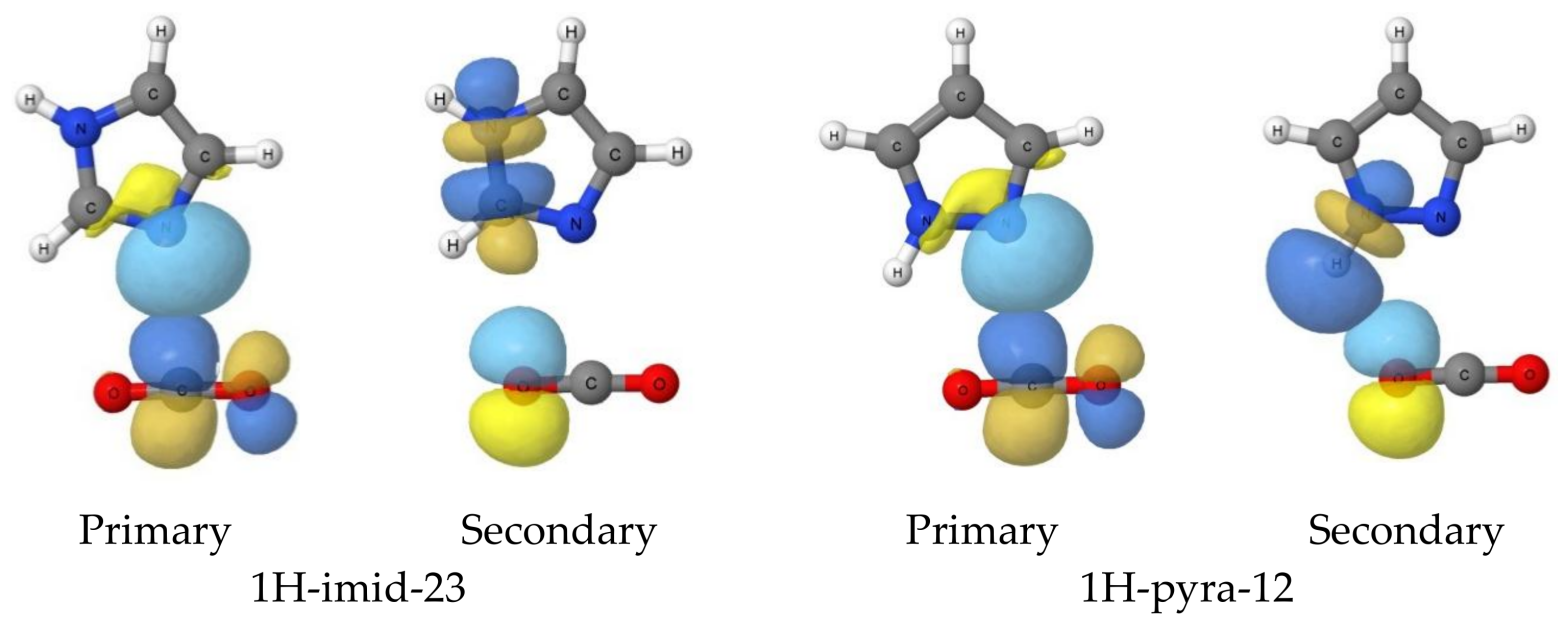
Orbital descriptions of the primary and secondary charge-transfer interactions in 1H-imid-23 and 1H-pyra-12.

**Figure 4:**
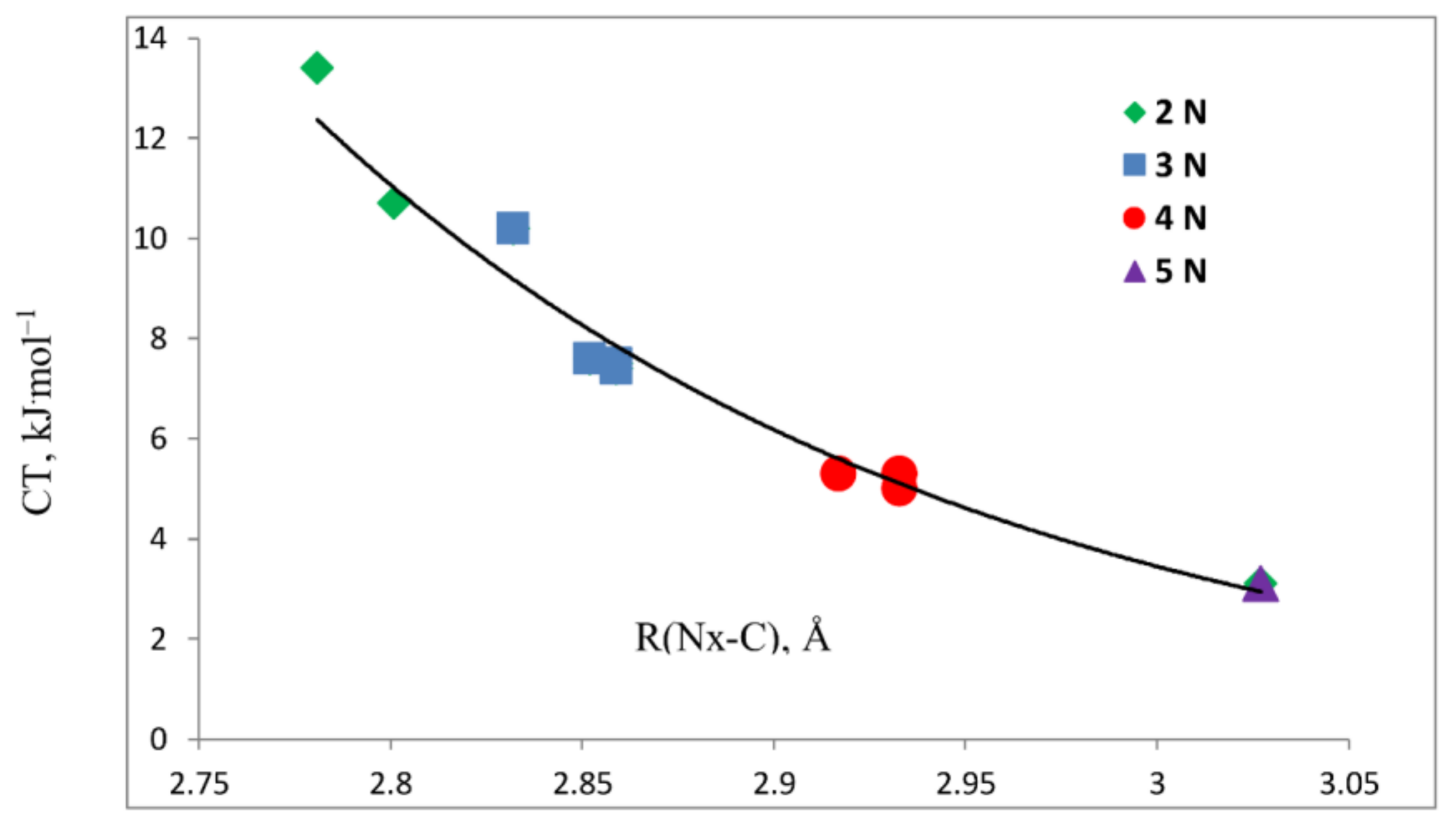
Primary Nx_lp_→σ*C-O charge-transfer energies versus the Nx-C distance. The legend indicates the number of nitrogen atoms in the azole ring.

The complexes 1H-imid-23 and 1H-124tri-45 are the two complexes that have a Cy-H bond adjacent to Nx. From Table 2 and Figure 2, it can be seen that although 1H-imid-23 has the shortest Nx-C distance, its binding energy is less than the binding energies of the planar tetrel-bonded complexes with pyrazole and the triazoles that have an Ny-H bond adjacent Nx. 1H-124tri-45 has the smallest binding energy among the planar tetrel-bonded complexes, even though its Nx-C distance is shorter than this distance in the complexes with the remaining triazoles, tetrazole and pentazole. The Cy-O’ distances in these two complexes are long, and the H-Cy-O’ angles are about 57°. These data suggest that Cy-H does not significantly interact with O’. This is consistent with the primary and secondary charge-transfer energies of these two complexes which are also reported in Table 2. Primary refers to the interaction associated with the tetrel bond. Figure 3 provides an orbital representation of the primary charge-transfer interaction in 1H-imid-23. The Nx lone pair donates charge to the σ antibonding C-O orbital in both 1H-imid-23 and 1H-124-tri-45. The σ antibonding C-O’ orbital is the local in-plane C-O π* orbital of CO_2_. The charge-transfer energies in these two complexes are greater than they are in the other planar tetrel-bonded complexes, except for 1H-pyra-12. The plot of Figure 4 shows that the primary charge-transfer energies correlate exponentially with the Nx-C distance, with a correlation coefficient of 0.978, and that these energies decrease as the number of nitrogen atoms in the ring increases. Thus, the charge-transfer energies reflect the relative strengths of the tetrel bonds in these complexes. The secondary charge-transfer energy in 1H-imid-23, which is also depicted in Figure 3, indicates that there is no Cy-H-O’ interaction in this complex. Thus, it is reasonable to conclude that 1H-imid-23 and 1H-124tri-45 are stabilized solely by tetrel bonds.

The second group of planar complexes is also stabilized by Nx···C tetrel bonds and by a secondary interaction between Ny-H and O’ and have Nx-C distances that are shorter than Ny-O’ distances. This set is composed of complexes with pyrazole and the triazoles. As evident from Figure 2, they have the largest binding energies which a range from 20.3 to 22.7 kJ·mol^−1^ and Nx-C distances between 2.801 and 2.859 Å. The secondary interaction between Ny-H and O’ may be described as a distorted hydrogen bond with H-Ny-O’ angles of about 40°. Nevertheless, the interaction between Ny-H and O’ must play a role in stabilizing these complexes, since the binding energies of the complexes with the triazoles are greater than the binding energy of 1H-imid-23, which has a much shorter Nx-C distance and no stabilizing secondary interaction. Figure 3 provides an orbital description of the primary and secondary charge-transfer interactions in 1H-pyra-12. The primary charge-transfer is Nx_lp_→σ*C-O, with charge-transfer energies of 10.7 kJ·mol^−1^ for 1H-pyra-12 and about 7.5 kJ·mol^−1^ for the complexes with the triazoles. These charge-transfer energies are greater than the energies of secondary back-donations of charge O’_lp_→σ*Ny-H associated with the distorted hydrogen bond, which are 6 kJ·mol^−1^ for 1H-pyra-12 and about 2 kJ·mol^−1^ for the complexes with the triazoles. These data suggest that the tetrel bond is primarily responsible for the large binding energies of these complexes, with the Ny-H-O’ interaction playing a secondary role.

The final group of planar complexes with tetrel bonds and Ny-H-O’ interactions includes complexes of CO_2_ with tetrazole and pentazole, which have Ny-O’ distances that are shorter than Nx-C distances, as evident from Figure 2. These four complexes have the smallest binding energies, and the longest Nx-C distances. While the Nx-C and Ny-O’ distances are similar for the tetrazole complexes, they are very different for 1H-pent-12. It may well be that the Ny-H-O’ interaction in the latter complex is the stronger interaction. This interaction could be described as a distorted Ny-H···O’ hydrogen bond, with H-Ny-O’ angles of about 40°. The primary charge transfer associated with the tetrel bond arises from electron donation from Nx to the σ antibonding C-O orbital, while the secondary charge transfer involves back-donation of charge from O’ to the antibonding σ Ny-H orbital. The secondary charge-transfer energy is greater than the primary charge transfer energy in 2H-tet-23 and 1H-pent-12. These data are consistent with the increased importance of the Ny-H-O’ interaction in these complexes. In all of the planar complexes with Ny-H bonds interacting with O’, the Nx-C and Ny-O’ distances are the best compromise to produce a stable equilibrium complex.

**Figure 5:**
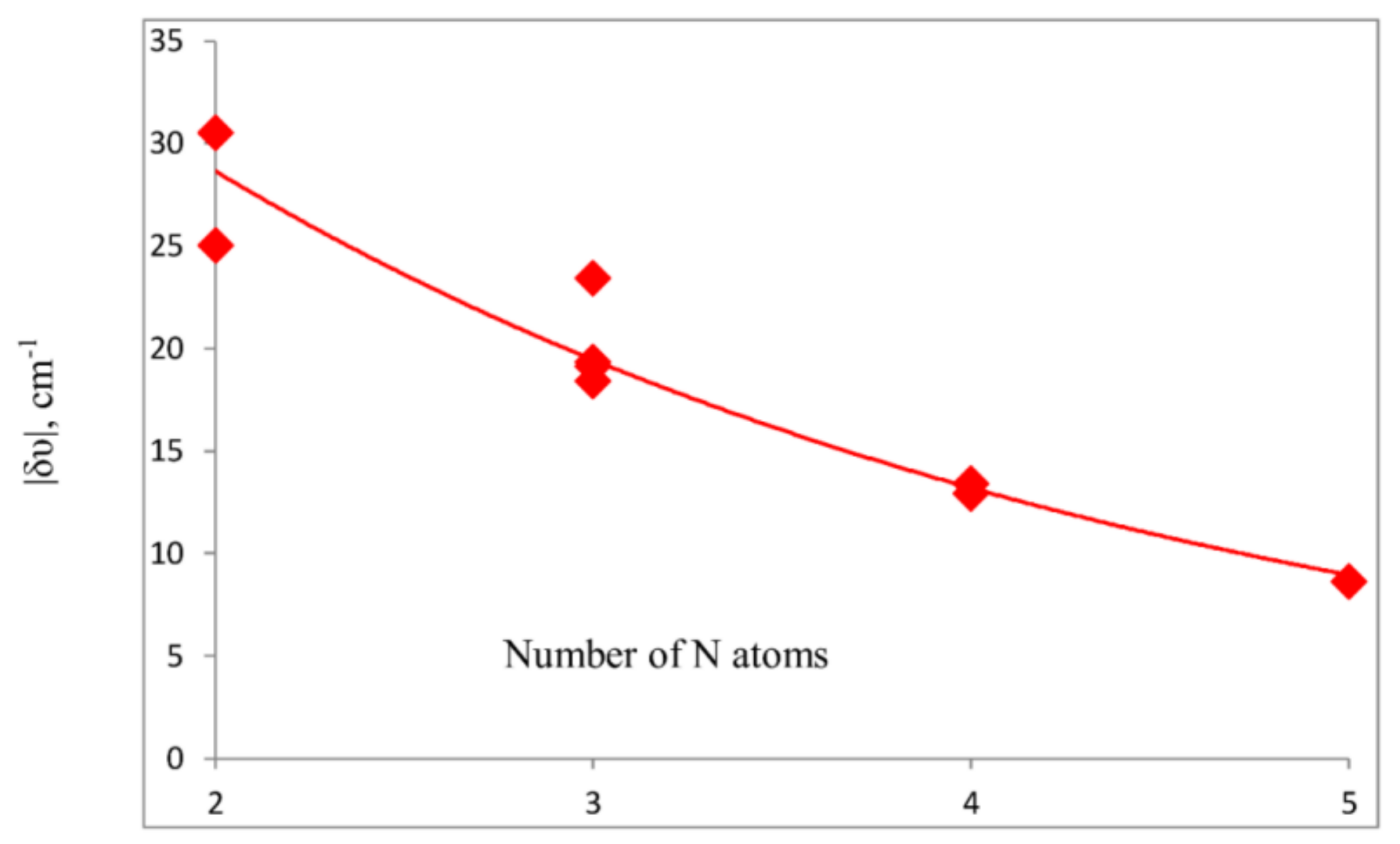
Absolute value of the change in the CO_2_ in-plane bending frequency upon complex formation versus the number of nitrogen atoms in the azole ring.

Harmonic symmetric and asymmetric stretching and in-plane and out-of-plane bending frequencies of isolated CO_2_ and of CO_2_ in planar tetrel-bonded complexes are reported in Table S2 of the Supporting Information. The symmetric and asymmetric stretching frequencies and the out-of-plane bending frequencies of CO_2_ change by less than 5 cm^−1^ upon complex formation. It is the in-plane CO_2_ bending frequency that is most sensitive to complexation, since changes in this frequency most directly affect both the Nx···C tetrel bond and the Ny-H···O interaction. This frequency decreases by 9 to 31 cm^−1^ in the complexes, as evident from Table 3. Figure 5 illustrates that the in-plane bending frequency of the planar tetrel-bonded complexes decreases as the number of nitrogen atoms in the azole ring increases. The correlation coefficient of the exponential trend line is 0.951. It is interesting to note that the Ny-H stretching frequency is also changed by complex formation, thereby giving another property that supports the importance of the Ny-H-O’ interaction in these complexes. This frequency is red-shifted by 22 to 30 cm^−1^ upon complexation, as evident from the plot of Figure S1 of the Supporting Information.

Table 3 also reports the one-bond coupling constants ^1t^J(Nx-C) across the tetrel bonds and J(Ny-O’) for the planar complexes. Only the FC term contributes to ^1t^J(Nx-C), and this term has values between 0.0 and 0.6 Hz. These small values may be attributed to the nature of the FC term, which depends on s electron densities in the ground and excited states of the coupled nuclei. Since the tetrel bond basically forms through the π system of CO_2_, there is little s-electron density at C in the direction of the Nx-C bond. Yet, despite the small values, ^1t^J(Nx-C) exhibits a second-order correlation with the Nx-C distance, with a correlation coefficient of 0.978. A plot of ^1t^J(Nx-C) versus the Nx-C distance is included as Figure S2 of the Supporting Information. Values of J(Ny-O’) are reported in Table 2 and are also small, ranging from 0.8 to 1.5 Hz. These are plotted against the Ny-O’ distance in Figure S3 of the Supporting Information. The correlation coefficient of the second-order trend line is not as good, with a value of 0.877. However, what is most interesting is a comparison of Figures S2 and S3. It is evident that as the number of N atoms in the azole ring increases, ^1t^J(Nx-C) decreases because the Nx-C distance increases, but J(Ny-O’) increases because the Ny-O’ distance decreases.

### 3.3. Perpendicular Complexes Stabilized by Tetrel Bonds

**Figure 6:**
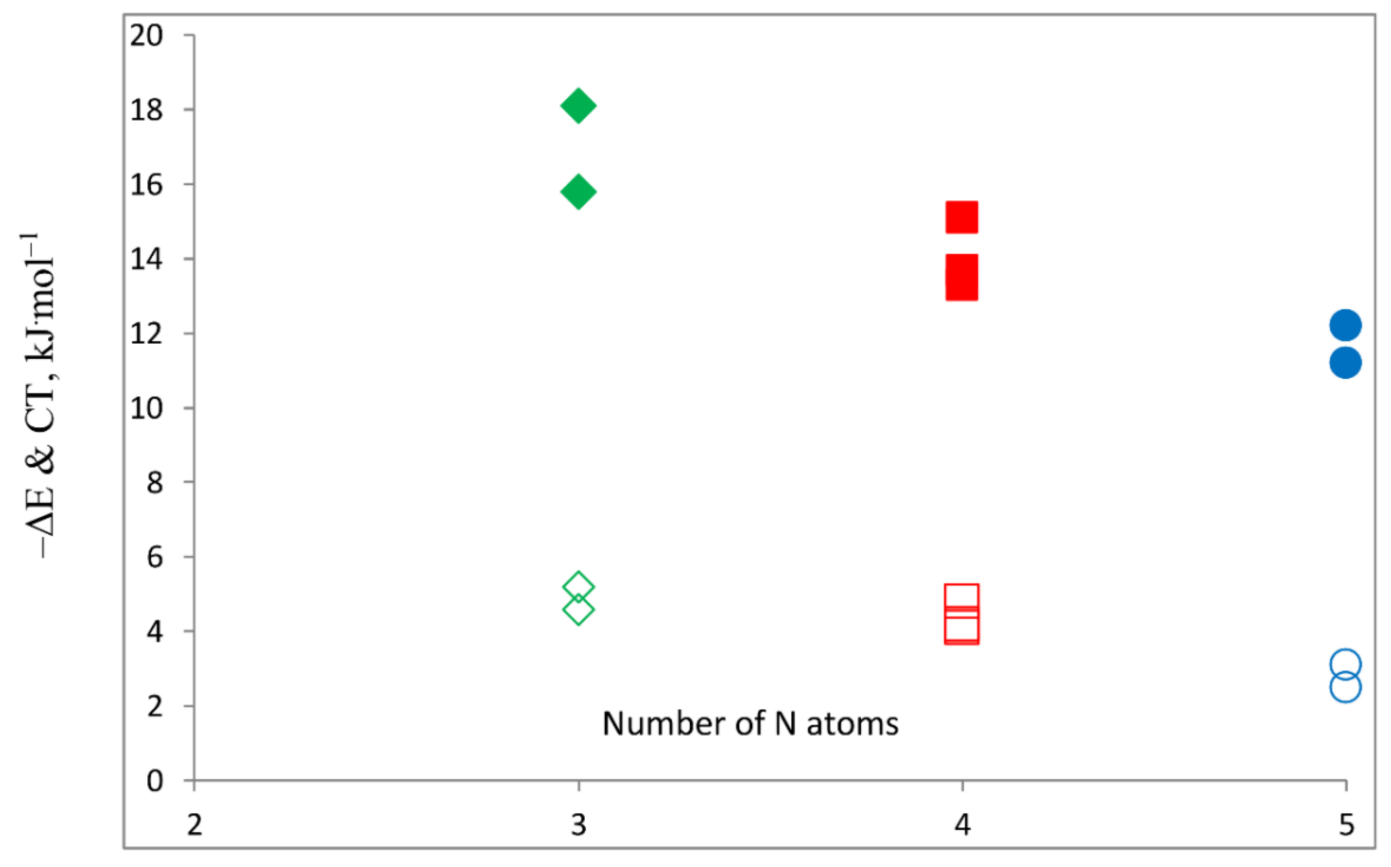
Binding energies (solid symbols) and charge-transfer energies (open symbols) versus the number of N atoms in the azole rings.

Table S3 of the Supporting Information provides the structures, total energies and molecular graphs of the perpendicular tetrel-bonded complexes, and Figure 1 illustrates the structure of 4H-124tri-12p, which has C_2v_ symmetry. Table 4 reports the binding energies, charge-transfer energies and Nx-C and Ny-C distances of these complexes. The most stable 4H-124tri-12p complex has a binding energy of 18.1 kJ·mol^−1^ and N1-C and N2-C distances of 2.922 Å. The least stable complex is 1H-pent-23p, which has a binding energy of 11.2 kJ·mol^−1^ and N3-C and N2-C distances of 2.939 and 3.113 Å, respectively. Since there are two different N-C distances in five of the seven perpendicular complexes, the binding energies have been plotted against the average of the Nx-C and Ny-C distances. This plot has an exponential trend line with a correlation coefficient of 0.916. However, what is more informative is Figure 6, in which the binding energies of the complexes are plotted against the number of nitrogen atoms in the ring. This plot illustrates very well that the binding energies of perpendicular complexes decrease as the number of nitrogen atoms in the ring increases.

Charge-transfer energies for the perpendicular complexes are also reported in Table 4. These arise from electron donation from the lone pairs on Nx and Ny to the in-plane antibonding π* O-C-O orbital of CO_2_. The NBO method describes charge-transfer in these complexes as two charge-transfer interactions, and it is the sum that is reported in Table 3. The total charge-transfer energies vary from 2.5 kJ·mol^−1^ for 1H-pent-23p to 5.6 kJ·mol^−1^ for 4H-124tri-12p. Figure 6 presents a plot of the total charge-transfer energies versus the number of nitrogen atoms in the azole ring.

**Figure 7:**
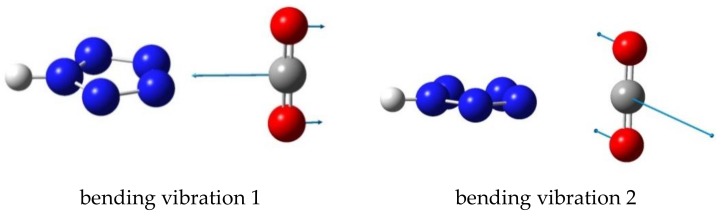
Representation of the two bending vibrations in the 1H-pent-34p complex.

In the perpendicular complexes, there are four vibrational frequencies associated with the CO_2_ molecule, two stretching and two bending vibrations, with the bending modes illustrated in Figure 7. Bending Vibration 1 may be roughly described as a bending motion that occurs in a plane perpendicular to the Nx-Ny bond, while Vibration 2 is a bending vibration in a plane that is parallel to the Nx-Ny bond. The stretching frequencies and bending frequency 2 are not very sensitive to complex formation, since the changes in these do not exceed 2.5 cm^−1^, but bending vibration 1 is sensitive to complexation. As evident from Table 4, this frequency decreases by about 14 cm^−1^ in complexes with the triazoles, by 11 or 12 cm^−1^ with the tetrazoles and by 8 or 9 cm^−1^ with pentazole. The decrease in this frequency exhibits a linear dependence on the number of nitrogen atoms, with a correlation coefficient of 0.971.

Coupling constants ^1t^J(Nx-C) and ^1t^J(Ny-C) have been evaluated for these complexes. Only the FC term contributes to these coupling constants. However, because the carbon atom of CO_2_ lies in the nodal plane, the computed FC terms are either 0.0 or 0.1 Hz.

### 3.4. Complexes Stabilized by Hydrogen Bonds

**Figure 8:**
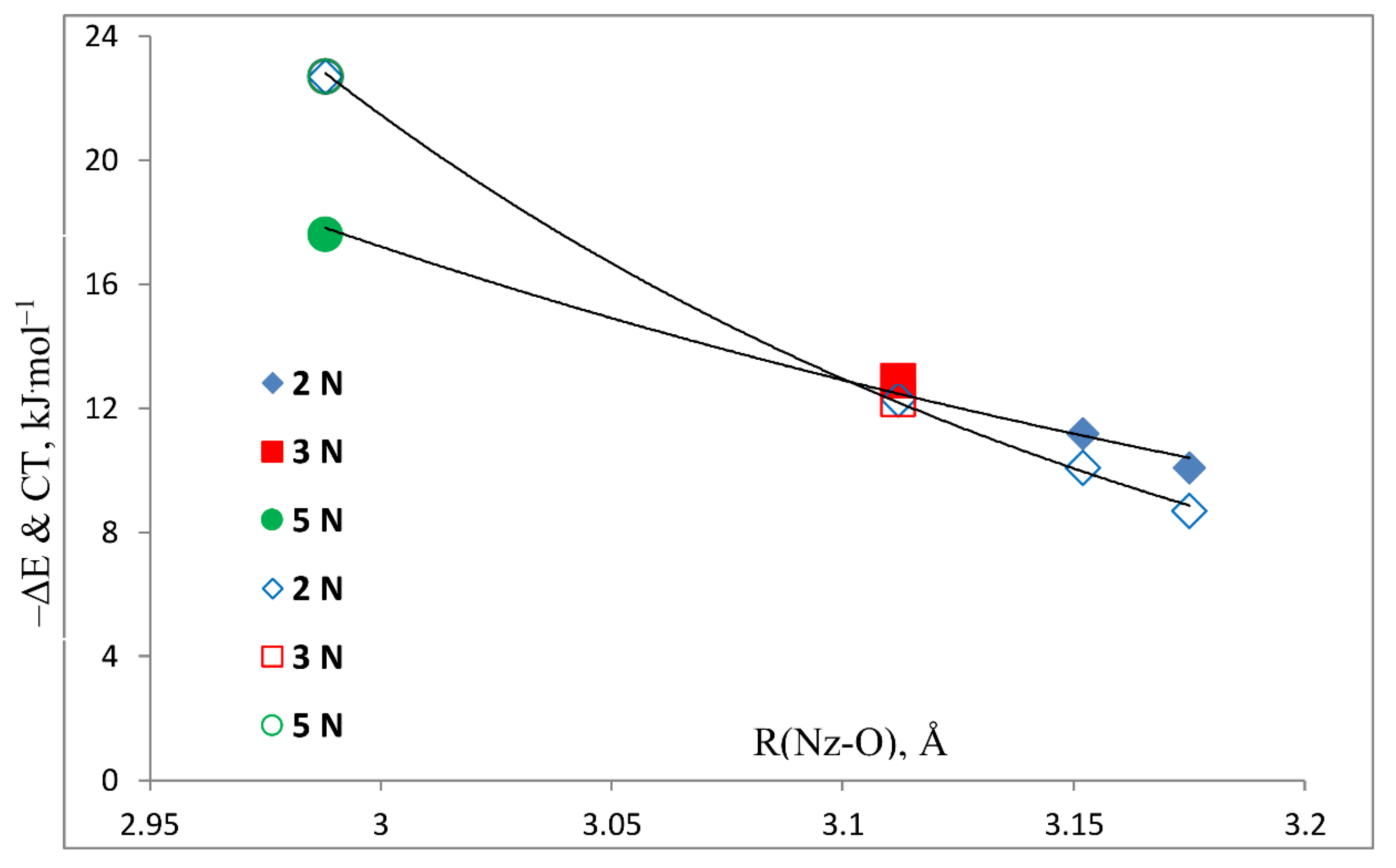
Binding energies (solid symbols) and charge-transfer energies (open symbols) versus the Nz-O distance.

There are only four CO_2_:azole complexes that are stabilized by Nz-H···O hydrogen bonds, 1H-pyrr, 1H-imid, 4H-124tri and 1H-pent. Table S4 of the Supporting Information provides their structures, total energies and molecular graphs, and 1H-imid is illustrated in Figure 1. Table 5 presents their binding energies, charge-transfer energies and Nz-O and Nz-H distances. The Nz-O distances are rather long, varying from 2.988 Å in 1H-pent to 3.175 Å in 1H-pyrr. The binding energies range from 10.1 kJ·mol^−1^ for 1H-pyrr to 17.6 kJ·mol^−1^ for 1H-pent, while the charge-transfer energies O_lp_→σ*Nz-H range from 8.7 to 22.7 kJ·mol^−1^ in these same two complexes. Plots of these two properties versus the Nz-O distance are given in Figure 8. The correlation coefficients of the exponential trend lines are 0.987 for the binding energies and 0.999 for the charge-transfer energies. From Table 5 and Figure 8, it is apparent that the binding energies and charge-transfer energies increase as the number of N atoms in the ring increases, unlike the tetrel-bonded complexes for which the binding energies tend to decrease as the number of nitrogen atoms increases. As the number of nitrogen atoms in the ring increases, the azole molecule becomes more acidic and Nz-H becomes a better proton donor, while the azole molecule becomes a weaker base and a poorer electron-pair donor.

Table 5 also reports IR and NMR spectroscopic data for these complexes including the change in the Nz-H stretching frequency upon complex formation and the two-bond NMR coupling constant ^2h^J(Nz-O) across the hydrogen bond. For a typical X-H···Y hydrogen bond, there is a red shift, that is a shift to lower energy of the X-H stretching frequency. The data of Table 5 show that this frequency decreases in the CO_2_:azole complexes relative to the corresponding isolated azole. The red shifts range from 5 to 47 cm^−1^ and increase as the number of nitrogen atoms in the ring and the Nz-H distance in the complexes increase. The linear trend line that relates the Nz-H stretching frequency to the Nz-H distance has a correlation coefficient of 0.996. The second spectroscopic property of interest is the NMR coupling constant ^2h^J(Nz-O) across the hydrogen bond. This coupling constant varies from 10.1 Hz in 1H-pyrr to 17.6 Hz in 1H-pent. The second-order trend line that relates ^2h^J(Nz-O) to the Nz-O distance has a correlation coefficient of 0.9997.

As evident from Table 5, there are only four CO_2_:azole complexes that are stabilized by hydrogen bonds. However, since there are ten different azoles, namely pyrrole, pyrazole, imidazole and pentazole, and two tautomers each of 1,2,3-triazole, 1,2,4-triazole and tetrazole, which differ in the position of the Nz-H bond, it would not be unreasonable to expect that there might be 10 hydrogen-bonded complexes. Why are there only four? Some insight into the answer to this question comes from the structures of the complexes 1H-pyrr, 1H-imid and 4H-124tri. In these, Nz-H is bonded to two C-H groups in the ring. Without an adjacent N atom with a lone pair of electrons available to form a tetrel bond, only an essentially linear Nz-H···O hydrogen bond can form. In contrast, pentazole does have N atoms with lone-pairs adjacent to Nz-H, and it does form one planar tetrel-bonded complex. However, the basicity of the azoles decreases and the acidity increases as the number of N atoms in the ring increases [52]. As a result, pentazole also forms a hydrogen-bonded complex with CO_2_, and it is the most stable among these complexes.

## 4. Conclusions

Ab initio MP2/aug’-cc-pVTZ calculations have been performed to investigate complexes of CO_2_ with the azoles pyrrole, pyrazole, imidazole, 1,2,3- and 1,2,4-triazole, tetrazole and pentazole. The results of these calculations support the following statements.

1. Three types of complexes have been found on the potential surfaces. These include ten complexes stabilized by tetrel bonds in which the azole molecule lies in the symmetry plane of the complex and seven complexes also stabilized by tetrel bonds, but which have the azole molecule perpendicular to the symmetry plane. In addition, there are four hydrogen-bonded complexes.
2. The ten complexes stabilized by tetrel bonds that have the azole molecule in the symmetry plane of the complex have some common characteristics.
  - Those complexes that have an Ny-H group bonded to Nx are stabilized primarily by Nx···C tetrel bonds and by a secondary interaction between Ny-H and O’, which assumes increased importance as the number of N atoms in the ring increases.
  - The binding energies of the planar complexes do not correlate with the Nx-C distance, but the primary charge-transfer energies do.
  - The IR in-plane bending frequency of CO_2_ is most sensitive to complex formation. The change in this frequency decreases as the number of N atoms increases. NMR spin-spin coupling constants ^1t^J(Nx-C), which are FC dominated, are less than 1 Hz, since there is little s-electron density at C in the direction of the tetrel bond.
3. There are seven perpendicular tetrel-bonded complexes that arise when there are two adjacent N atoms in the ring, and each has a lone pair of electrons.
  - The binding energies of perpendicular complexes decrease as the number of nitrogen atoms in the ring decreases.
  - The IR bending mode of CO_2_ that moves the C atom toward and away from the Nx-Ny bond is most sensitive to complex formation. The change in its frequency upon complex formation decreases in the order triazole > tetrazole > pentazole.
  - The NMR coupling constants ^1t^J(Nx-C) and ^1t^J(Ny-C) are negligibly small since there is a node at C in the complex symmetry plane.
4. The four hydrogen-bonded complexes involve pyrrole, imidazole, 1,2,4-triazole and pentazole. Three of these form when the ring Nz-H is bonded to two C-H groups, thereby eliminating the possibility of tetrel-bond formation. The fourth forms with pentazole, which is the strongest acid.
  - The binding energies of these complexes and their charge-transfer energies increase as the number of N atoms in the ring increases.
  - Hydrogen bonding produces a red-shift of the IR Nz-H stretching band. The magnitude of the red shift increases as the number of N atoms in the ring increases.
  - The NMR coupling constant ^2h^J(Nz-O) across the hydrogen bond increases as the number of nitrogen atoms in the ring increases.

## Figures and Tables

**Table 1:** Names of CO_2_:azole complexes, their binding energies (−ΔE, kJ·mol^−1^) and symmetries.

Azole	Complex	−ΔE	Sym
pyrrole	1H-pyrr	10.1	*C*_2*v*_
pyrazole	1H-pyra-12	22.7	*C_s_*
imidazole	1H-imid-23	19.9	*C_s_*
	1H-imid	11.2	*C_s_*
triazoles	1H-123tri-12	21.8	*C_s_*
	2H-123tri-12	20.3	*C_s_*
	1H-123tri-23p	15.8	*C_s_*
	1H-124tri-12	21.0	*C_s_*
	4H-124tri-12p	18.1	*C*_2*v*_
	1H-124tri-45	17.9	*C_s_*
	4H-124tri	12.9	*C*_2*v*_
tetrazoles	1H-tet-12	20.3	*C_s_*
	2H-tet-23	19.8	*C_s_*
	2H-tet-12	18.9	*C_s_*
	1H-tet-34p	15.1	*C_s_*
	2H-tet-34p	13.7	*C_s_*
	1H-tet-23p	13.3	*C_s_*
pentazole	1H-pent-12	19.0	*C_s_*
	1H-pent	17.6	*C*_2*v*_
	1H-pent-34p	12.2	*C*_2*v*_
	1H-pent-23p	11.2	*C_s_*

**Table 2:** Binding energies (−ΔE) and charge-transfer energies (kJ·mol^−1^), Nx-C, Ny-O’, Cy-O’ and H-O’ distances (R, Å) for planar CO_2_:azole complexes stabilized by Nx···C tetrel bonds.

Azole	Complex	−ΔE	R(Nx-C)	R(Ny-O’); R(Cy-O’) ^a^	R(NyH-O’); R(CyH-O’) ^a,b^	Primary CT ^c^	Secondary CT ^d^
pyrazole	1H-pyra-12	22.7	N2: 2.801	N1: 2.939	2.285	10.7	2.0
imidazole	1H-imid-23	19.9	N3: 2.781	C2: 3.171	*2.732*	13.4	1.0 ^d^
triazoles	1H-123tri-12	21.8	N2: 2.852	N1: 2.918	2.250	7.6	6.1
	2H-123tri-12	20.3	N1: 2.859	N2: 2.936	2.298	7.5	2.1
	1H-124tri-12	21.0	N2: 2.859	N1: 2.933	2.275	7.4	2.3
	1H-124tri-45	17.9	N4: 2.832	C5: 3.156	*2.707*	10.2	1.1 ^e^
tetrazoles	1H-tet-12	20.3	N2: 2.933	N1: 2.094	2.222	5.0	4.5
	2H-tet-23	19.8	N3: 2.917	N2: 2.904	2.252	5.3	6.3
	2H-tet-12	18.9	N1: 2.933	N2: 2.917	2.264	5.3	2.6
pentazole	1H-pent-12	19.0	N2: 3.027	N1: 2.878	2.197	3.1	4.7

^a^ Ny-H or Cy-H is adjacent to Nx. ^b^ H-O’ distances involving Cy-H are given in italics. ^c^ Nx_lp_→σ*C-O. ^d^ O’_lp_→σ*Ny-H. ^e^ The charge-transfer is O’_lp_→σ*Cy-N1.

**Table 3:** CO_2_ in-plane bending frequencies (ν), changes in these frequencies upon complexation (δν, cm^−1^), and spin-spin coupling constants ^1t^J(Nx-C) and J(Ny-O’) (Hz) for planar CO_2_:azole complexes stabilized by Nx···C tetrel bonds.

Azole	Complex	ν ^a^	δν	^1t^J(Nx-C)	J(Ny-O’)
pyrazole	1H-pyra-12	634	−25.0	0.5	0.8
imidazole	1H-imid-23	628	−30.5	0.6	
triazoles	1H-123tri-12	640	−19.1	0.4	0.9
	2H-123tri-12	641	−18.4	0.3	1.0
	1H-124tri-12	640	−19.3	0.3	0.9
	1H-124tri-45	636	−23.4	0.4	
tetrazoles	1H-tet-12	646	−13.4	0.2	1.1
	2H-tet-23	646	−13.4	0.2	1.2
	2H-tet-12	646	−12.9	0.1	1.1
pentazoles	1H-pent-12	650	−8.6	0.0	1.5

^a^ The degenerate bending frequency of isolated CO_2_ is 659 cm^−1^.

**Table 4:** Binding energies (−ΔE) and charge-transfer energies (CT, kJ·mol^−1^), Nx-C and Ny-C distances (Å) and frequencies of bending vibration 1 (ν, cm^−1^) in perpendicular azole:CO_2_ complexes with tetrel bonds.

Azole	Complex	−ΔE	R(Nx-C); R(Ny-C) ^a^	CT ^b^	υ ^c,d^
triazoles	1H-123tri-23p	15.8	N2: 2.994; N3: 2.938	4.4	645
	4H-124tri-12p	18.1	N1: 2.922; N2: 2.922	5.6	644
tetrazoles	1H-tet-34p	15.1	N3: 2.959; N4: 2.981	4.9	648
	2H-tet-34p	13.7	N3: 3.021; N4: 2.967	4.2	647
	1H-tet-23p	13.3	N2: 3.088; N3:2.954	4.1	647
pentazoles	1H-pent-34p	12.2	N3: 3.016; N4: 3.016	3.0	651
	1H-pent-23p	11.2	N2: 3.113; N3: 2.939	2.5	650

^a^ Two electron-donor N atoms that form the tetrel bond. ^b^ The charge transfer is (Nx_lp_ + Ny_lp_)→π*O-C-O. In the natural bond orbital (NBO) scheme, this is the sum of two charge-transfer interactions. ^c^ The degenerate bending vibrational frequency of isolated CO_2_ is 659 cm^−1^. ^d^ The bending vibrations are illustrated in Figure 7.

**Table 5:** Binding energies (−ΔE) and charge-transfer energies (kJ·mol^−1^), Nz-O and Nz-H distances (Å), changes in Nz-H stretching frequencies (δν, cm^−1^) and spin-spin coupling constants ^2h^J(Nz-O) (Hz) for CO_2_:azole complexes stabilized by Nz-H···O hydrogen bonds.

Azole	Complex	−ΔE	R(Nz-O)	O_lp_→σ*Nz-H	^2h^J(Nz-O)	R(Nz-H)	Δν(Nz-H)
pyrrole	1H-pyrr	10.1	3.175	8.7	2.1	1.006	−5.1
imidazole	1H-imid	11.2	3.152	10.1	2.2	1.007	−8.8
124-triazole	4H-124tri	12.9	3.112	12.3	2.6	1.008	−17.1
pentazole	1H-pent	17.6	2.988	22.7	4.6	1.014	−47.0

## Supplementary Materials

Structures, total energies and molecular graphs of CO_2_:azole complexes; bending and stretching frequencies of CO_2_ in planar complexes; plots of stretching frequencies and coupling constants versus distances and/or the number of N atoms in the ring.
