# Supplementary material for: Complexes of CO2 with the Azoles: Tetrel Bonds, Hydrogen Bonds and Other Secondary Interactions
Source: Molecules. 2018 Apr 14;23(4):906. doi: 10.3390/molecules23040906 (PMC6017967; doi:10.3390/molecules23040906)
Supplement: Supplementary file 1 [file molecules-23-00906-s001.pdf]

Supporting Information for

Complexes of CO_2 with Azoles: Tetrel Bonds, Hydrogen Bonds, and Other Secondary Interactions

Janet E. Del Bene,[‡] José Elguero,[§] and Ibon Alkorta,[§]

[‡] Department of Chemistry, Youngstown State University, Youngstown, Ohio 44555 USA

[§] Instituto de Química Médica (IQM-CSIC), Juan de la Cierva, 3, E-28006 Madrid, Spain

Pgs. S2-S5 Table S1. Structures (Å), total energies (a.u.), and molecular graphs of tetrel-bonded planar CO_2:azole complexes

Pg. S6 Table S2. Bending and stretching frequencies (υ, cm^{-1}) of isolated CO_2 and of CO_2 in planar tetrel-bonded complexes

Pg. S7 Fig. S1. Fig. S1. Changes in Ny-H stretching frequencies in planar tetrel-bonded complexes versus the number of N atoms in the ring.

Pg. S8 Fig. S2. ^{1t}J(Nx-C) versus the Nx-C distance for planar tetrel-bonded complexes

Pg. S9 Fig. S3. J(Ny-O′) versus the Ny-O′ distance for planar tetrel-bonded complexes

Pgs. S10-S12 Table S3. Structures (Å), total energies (a.u.), and molecular graphs of tetrel-bonded perpendicular CO_2:azole complexes

Pgs. S13-S14 Table S4. Structures (Å), total energies (a.u.), and molecular graphs of hydrogen-bonded CO_2:azole complexes

Table S1. Structures (Å), total energies (a.u.), and molecular graphs of tetrel-bonded planar CO_2:azole complexes

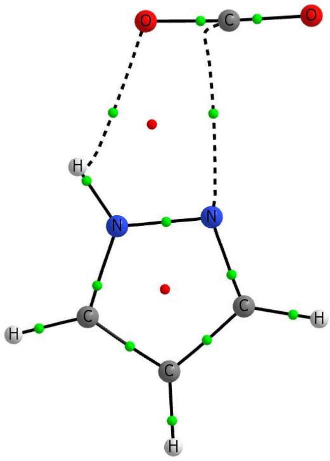	1H-pyra-12<br>MP2= -414.10610997 NIMAG= 0<br>C,3.2432998028,-2.1422600303,0.<br>O,3.6307835737,-1.0340604687,0.<br>O,2.9166154742,-3.2621442876,0.<br>N,0.7021901018,-0.9640695158,0.<br>N,1.0302062904,0.3354070945,0.<br>C,-0.6439824596,-0.9613821406,0.<br>C,-0.0412087389,1.1646314764,0.<br>C,-1.1572813018,0.3443387516,0.<br>H,-1.1707973373,-1.9006900132,0.<br>H,2.0094409995,0.5761665732,0.<br>H,0.0720257523,2.2350355869,0.<br>H,-2.1873646872,0.6541364138,0.
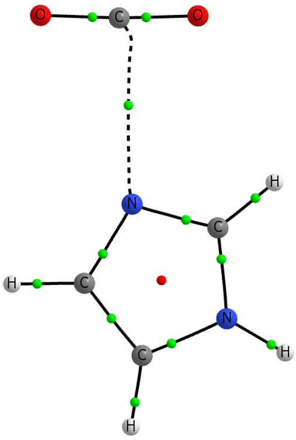	1H-imid-23<br>MP2= -414.12126121 NIMAG= 0<br>C,-2.4453954692,0.0163232368,0.<br>O,-2.2675842856,1.1746291193,0.<br>O,-2.6894879408,-1.1267362946,0.<br>N,2.2491339997,0.8053239583,0.<br>C,0.8926691015,0.9118824533,0.<br>N,0.3195650673,-0.2808900196,0.<br>C,1.3539713417,-1.1835220297,0.<br>C,2.5653507012,-0.5294157901,0.<br>H,1.1745793438,-2.245027426,0.<br>H,2.9031162869,1.5702957908,0.<br>H,0.3773681717,1.8574963297,0.<br>H,3.5821785919,-0.8795164983,0.
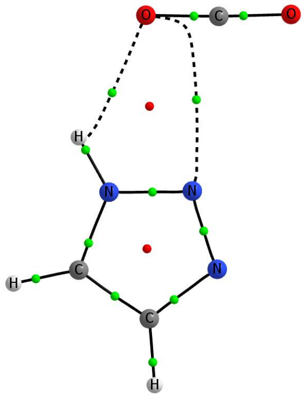	1H-123tri-12<br>MP2= -430.12853114 NIMAG= 0<br>C,3.2354213986,-2.1571904374,0.<br>O,3.5933671576,-1.0381236675,0.<br>O,2.9280363593,-3.2813946268,0.<br>N,1.0329529795,0.3621081374,0.<br>N,0.6621987315,-0.9282342658,0.<br>N,-0.663247646,-0.9445873824,0.<br>C,-0.0296672439,1.1948747201,0.<br>C,-1.1105631763,0.3361858398,0.<br>H,2.0213105768,0.5717940923,0.<br>H,0.0663769457,2.2654216211,0.<br>H,-2.1614612757,0.5649454228,0.

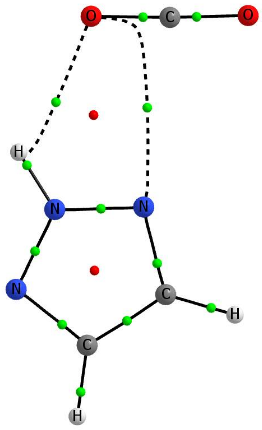	2H-123tri-12<br>MP2= -430.13530138 NIMAG= 0<br>C,3.2933550286,-2.1279297602,0.<br>O,3.6187390348,-1.0002137407,0.<br>O,3.0138948314,-3.2605141693,0.<br>N,0.9748720105,0.2773519743,0.<br>N,0.6617366904,-1.011310578,0.<br>N,-0.0256285276,1.1454985494,0.<br>C,-0.6874048453,-0.9857816272,0.<br>C,-1.1094346434,0.3418491912,0.<br>H,-1.2502820077,-1.9020668612,0.<br>H,1.9411763344,0.570916358,0.<br>H,-2.0991221886,0.7622745169,0.
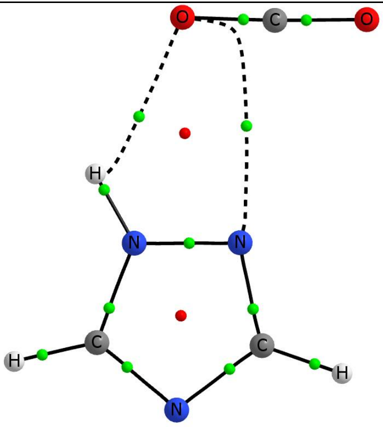	1H-124tri-12<br>MP2= -430.15368316 NIMAG= 0<br>C,3.2956578076,-2.1557483799,0.<br>O,3.6450728067,-1.0348497283,0.<br>O,2.9940721005,-3.2821757123,0.<br>N,0.6900264853,-0.9798470779,0.<br>N,1.0420816504,0.3174297227,0.<br>C,-0.6445872111,-0.9009989713,0.<br>N,-1.1449716982,0.3603477092,0.<br>C,-0.0493819263,1.1091472371,0.<br>H,-1.2570552949,-1.7859281956,0.<br>H,2.0219553427,0.5590328966,0.<br>H,-0.0015779228,2.1845635195,0.
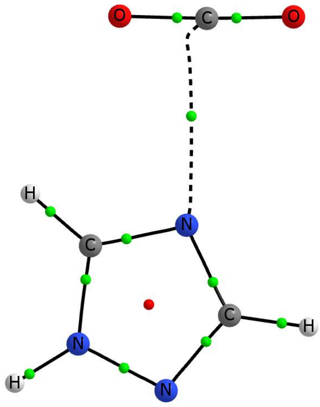	1H-124tri-45<br>MP2= -430.15248122 NIMAG= 0<br>C,-2.4390824651,0.1933198228,0.<br>O,-2.1542061596,1.3301570155,0.<br>O,-2.7737856244,-0.9260536728,0.<br>N,2.5962715301,-0.6407984787,0.<br>N,2.3018192463,0.671289904,0.<br>C,1.3786340551,-1.1897783443,0.<br>N,0.3491978711,-0.3037298721,0.<br>C,0.9680871005,0.8699880018,0.<br>H,1.244751248,-2.2575136001,0.<br>H,3.0462500584,1.3499888968,0.<br>H,0.5041797496,1.8415024872,0.

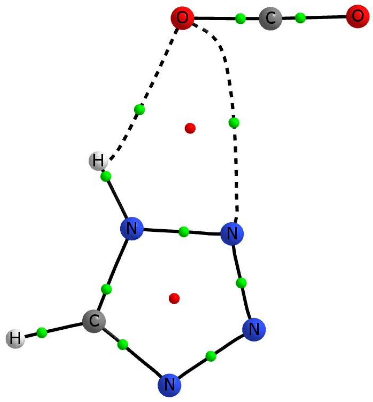	1H-tet-12<br>MP2= -446.15444402 NIMAG= 0<br>C,3.2763631041,-2.1760388934,0.<br>O,3.5881277109,-1.0430472973,0.<br>O,3.0018257053,-3.3082658322,0.<br>N,1.0388684985,0.348231601,0.<br>N,0.6241943203,-0.9238050043,0.<br>N,-0.6924350418,-0.870609022,0.<br>N,-1.1201990638,0.4143240639,0.<br>C,-0.0281345497,1.162353954,0.<br>H,2.0307701632,0.5414588984,0.<br>H,0.016805243,2.2362515521,0.
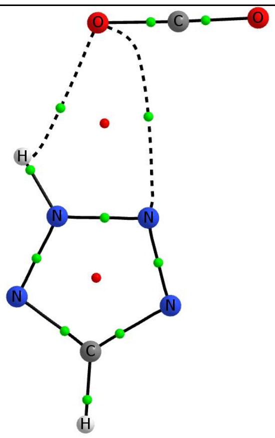	2H-tet-23<br>MP2= -446.15987382 NIMAG= 0<br>C,3.2905608608,-2.1404527626,0.<br>O,3.5735214019,-1.0003140078,0.<br>O,3.0451932882,-3.2797936329,0.<br>N,-0.0126099953,1.1730058555,0.<br>N,0.9776427463,0.3023151624,0.<br>N,0.6149138717,-0.9782236414,0.<br>N,-0.7133704827,-0.9583019294,0.<br>C,-1.0656258054,0.3405637443,0.<br>H,1.9548410166,0.5656173052,0.<br>H,-2.082883192,0.6877246168,0.
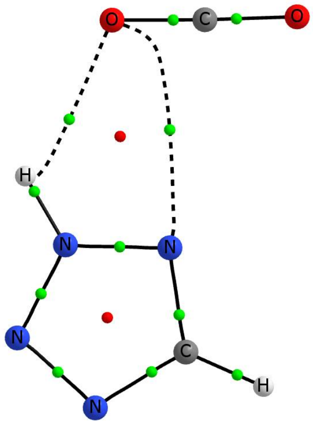	2H-tet-12<br>MP2= -446.15951455 NIMAG= 0<br>C,3.3580551859,-2.129904022,0.<br>O,3.6220894099,-0.9859544262,0.<br>O,3.1271917179,-3.2728431277,0.<br>N,0.6354678058,-1.04001045,0.<br>N,0.9733939654,0.2358625691,0.<br>N,-0.0392023779,1.0970737466,0.<br>N,-1.1308879537,0.339651276,0.<br>C,-0.7026366869,-0.9357903057,0.<br>H,-1.3540677703,-1.7907932311,0.<br>H,1.941620594,0.530507301,0.

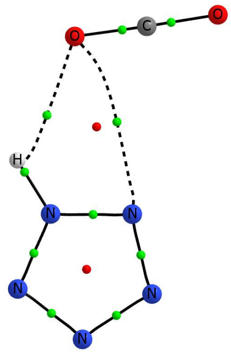	1H-pent-12<br>MP2= -462.16526638 NIMAG= 0<br>C,2.6982918766,0.1273269629,0.<br>O,2.8979702423,-1.0306504994,0.<br>O,2.523769481,1.2791039842,0.<br>N,0.3056582775,-2.2812947495,0.<br>N,-0.1001285387,-1.0258783962,0.<br>N,-0.6782788349,-3.1584512782,0.<br>N,-1.417569983,-1.1179307823,0.<br>N,-1.7678100151,-2.4112852975,0.<br>H,1.2888743343,-2.527052714,0.

Table S2. Bending and stretching frequencies (υ, cm^{-1}) of isolated CO_2 and of CO_2 in planar tetrel-bonded complexes

		out-of-plane bending	in-plane bending	Symmetric stretch	Asymmetric stretch
	CO_2	659	659	1326	2401
Azole	Complex				
pyrazole	1H-pyra-12	661	634	1327	2402
imidazole	1H-imid-23	664	628	1327	2400
triazoles	1H-123tri-12	660	640	1327	2404
	2H-123tri-12	660	641	1327	2404
	1H-124tri-12	660	640	1328	2404
	1H-124tri-45	663	636	1328	2402
tetrazoles	1H-tet-12	658	646	1327	2405
	2H-tet-23	659	646	1327	2405
	2H-tet-12	659	646	1328	2405
pentazole	1H-pent-12	657	650	1328	2406

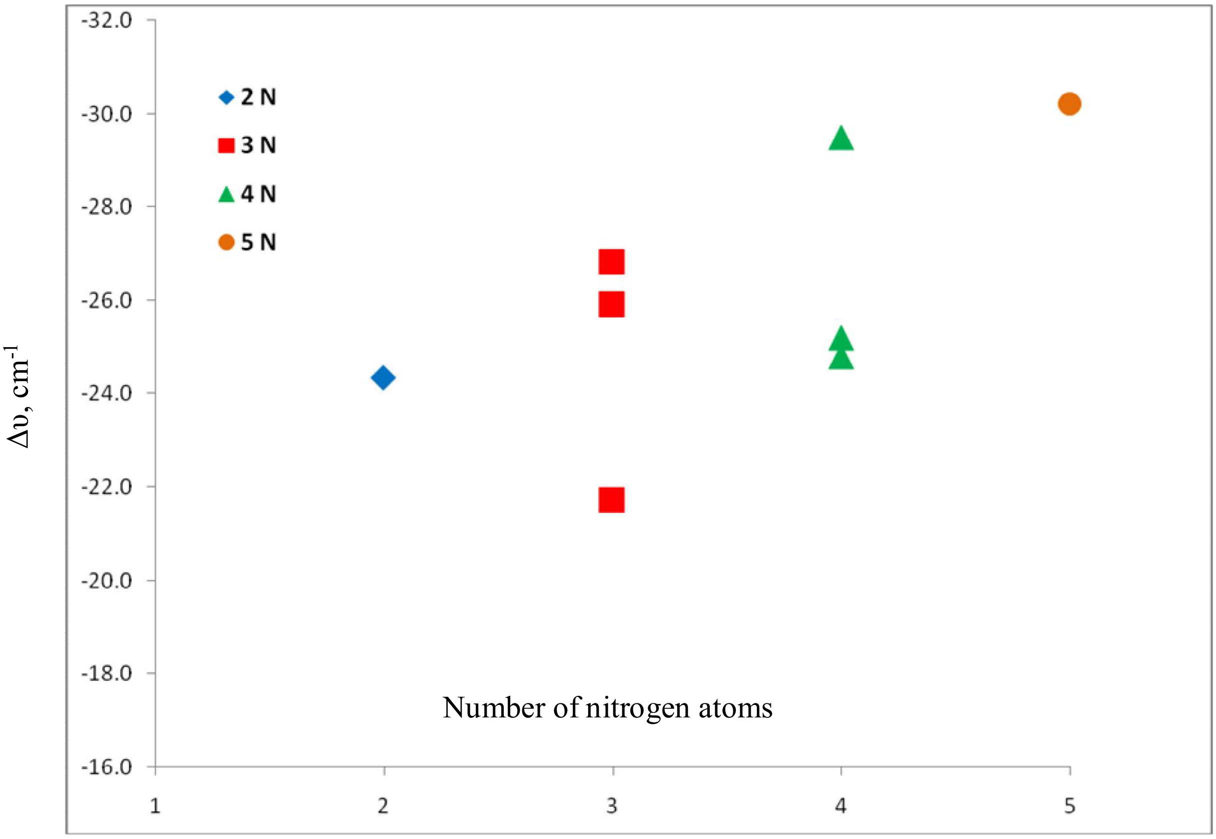

Fig. S1. Changes in Ny-H stretching frequencies in planar tetrel-bonded complexes versus the number of N atoms in the ring.

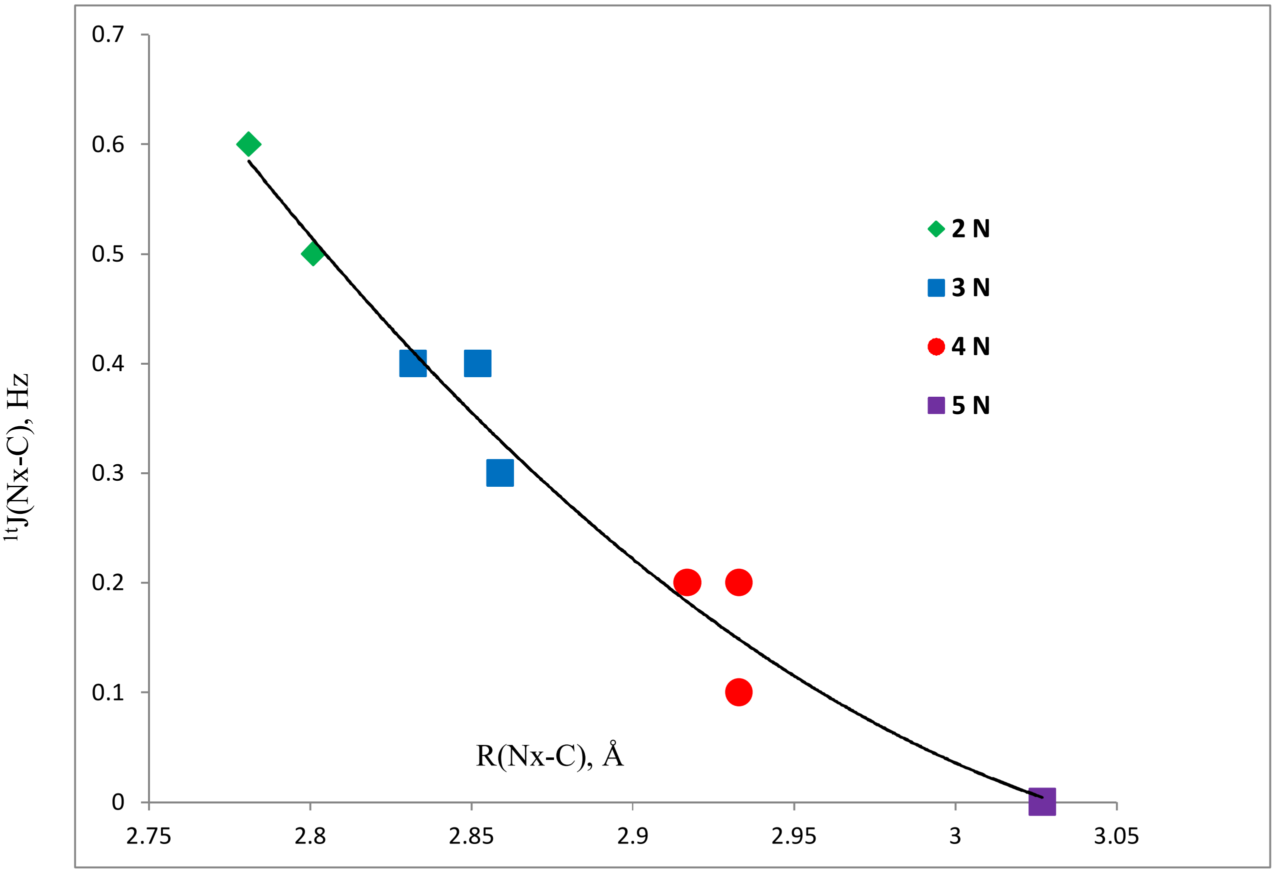

Fig. S2. ${}^{1t}J$(Nx-C) versus the Nx-C distance for planar tetrel-bonded complexes

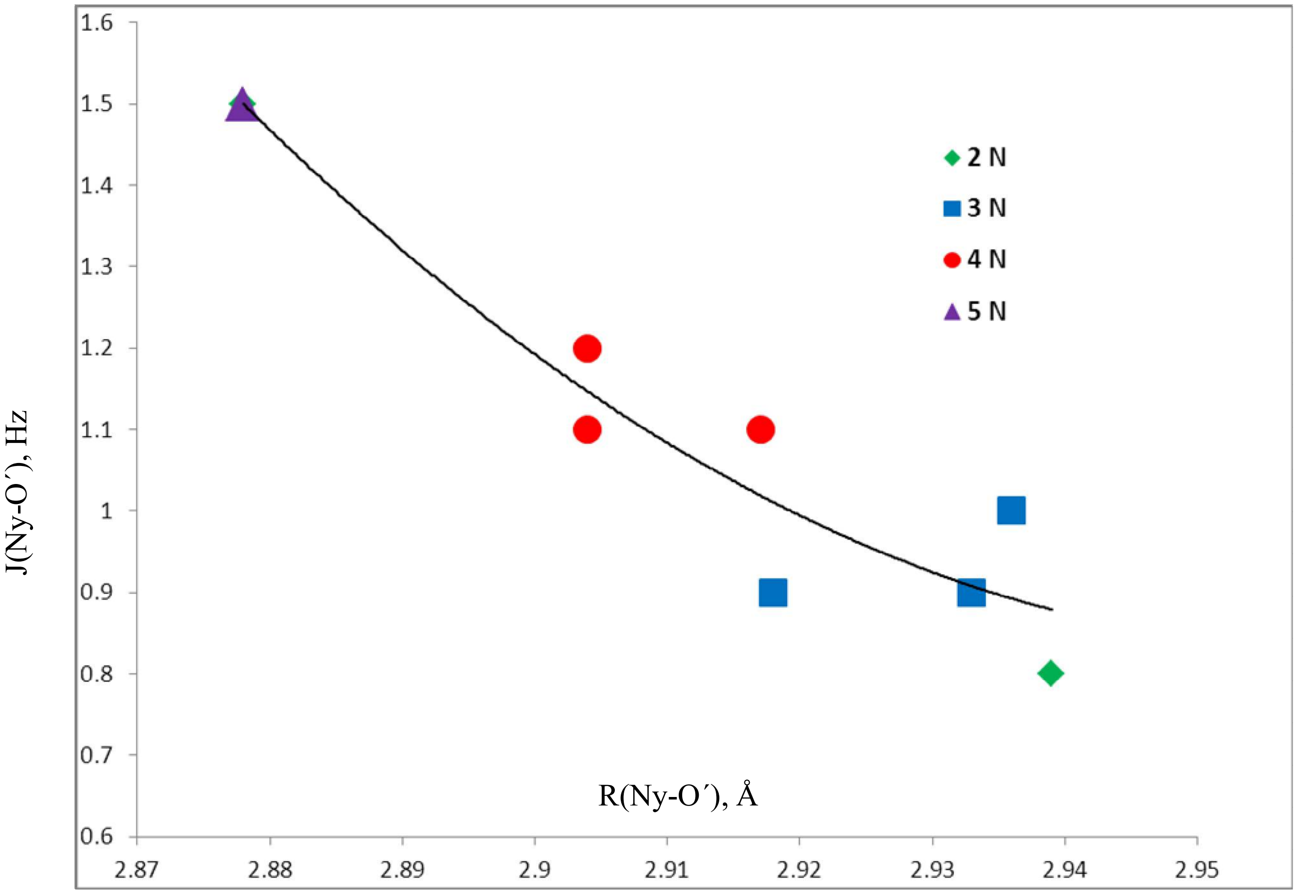

Fig. S3. J(Ny-O´) versus the Ny-O´ distance for planar tetrel-bonded complexes

Table S3. Structures (Å), total energies (a.u.), and molecular graphs of tetrel-bonded perpendicular CO_2:azole complexes

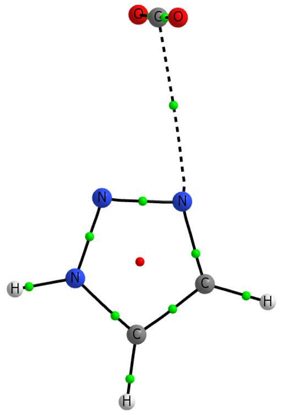	1H-123tri-23p<br>MP2= -430.12623017 NIMAG= 0<br>C,0.0193666139,0.,-3.9192408243<br>O,0.016563709,1.1699533762,-3.9394666568<br>O,0.016563709,-1.1699533762,-3.9394666568<br>N,-1.0624660736,0.,0.2788888579<br>N,-0.7087456306,0.,-1.015231868<br>N,0.6165036478,0.,-1.0424757459<br>C,1.0799169353,0.,0.2322498716<br>C,0.008901476,0.,1.1021734811<br>H,-0.0767248975,0.,2.1735109375<br>H,2.1332421598,0.,0.4489583011<br>H,-2.0431216492,0.,0.5143883927
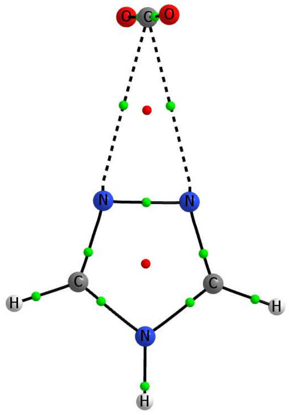	4H-124tri-12p<br>MP2= -430.14200266 NIMAG= 0<br>C,0.,0.,-3.8796093137<br>O,0.,1.169850826,-3.9024000548<br>O,0.,-1.169850826,-3.9024000548<br>N,0.6893925965,0.,-1.0400671923<br>N,-0.6893925965,0.,-1.0400671923<br>C,1.0807570502,0.,0.2188521624<br>C,-1.0807570502,0.,0.2188521624<br>N,0.,0.,1.0451520233<br>H,0.,0.,2.0519045151<br>H,2.1004401687,0.,0.562035506<br>H,-2.1004401687,0.,0.562035506
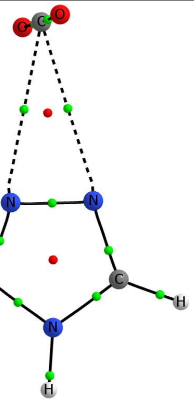	1H-tet-34p<br>MP2= -446.15242792 NIMAG= 0<br>C,-0.0400504806,0.,-3.8996071895<br>O,-0.039145282,1.1699429134,-3.9161980066<br>O,-0.039145282,-1.1699429134,-3.9161980066<br>N,0.0072175728,0.,1.014263033<br>N,-1.0815458463,0.,0.2349101273<br>N,-0.6282909974,0.,-0.9994222547<br>N,0.727757013,0.,-1.0189271573<br>C,1.1094530328,0.,0.2467252077<br>H,-0.0918373611,0.,2.0179021338<br>H,2.1187092911,0.,0.6164518029

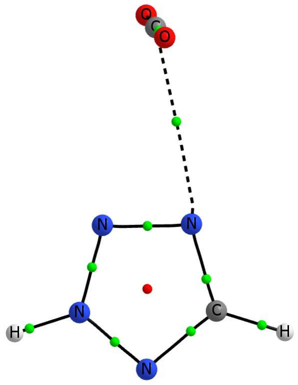	2H-tet-34p<br>MP2= -446.15752637 NIMAG= 0<br>C,0.0151807066,0.,-3.9596903595<br>O,0.0129142903,1.1700251958,-3.9749182619<br>O,0.0129142903,-1.1700251958,-3.9749182619<br>N,0.0105789243,0.,1.1000260199<br>N,-1.0126557374,0.,0.2674525119<br>N,-0.7038540642,0.,-1.0257470604<br>N,0.62432727,0.,-1.0558224527<br>C,1.0295867493,0.,0.2270837799<br>H,2.0598798307,0.,0.533161686<br>H,-1.9721473495,0.,0.5841495388
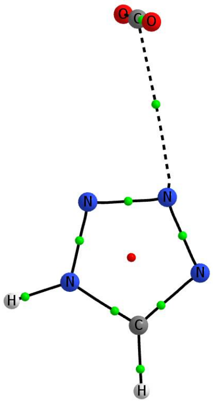	1H-tet-23p<br>MP2= -446.15175141 NIMAG= 0<br>C,0.0760410761,0.,-3.9449944864<br>O,0.0720640527,1.1700205784,-3.9602107625<br>O,0.0720640527,-1.1700205784,-3.9602107625<br>N,-1.110834781,0.,0.3168729464<br>N,-0.7948889615,0.,-0.9827408927<br>N,0.5215616004,0.,-1.024388667<br>N,1.0462790547,0.,0.2236160438<br>C,0.0142611853,0.,1.0511044386<br>H,0.0487983432,0.,2.1253695509<br>H,-2.0785877732,0.,0.600912371
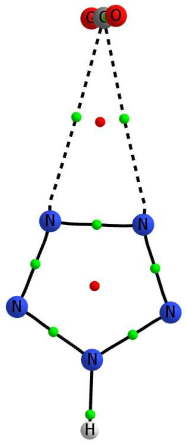	1H-pent-34p<br>MP2= -462.16267680 NIMAG= 0<br>C,0.,0.,-3.9199249944<br>O,0.,1.1700468519,-3.9304125838<br>O,0.,-1.1700468519,-3.9304125838<br>N,0.6704093968,0.,-0.9793025741<br>N,-0.6704093968,0.,-0.9793025741<br>N,1.1069848368,0.,0.2653755437<br>N,-1.1069848368,0.,0.2653755437<br>N,0.,0.,0.9836936856<br>H,0.,0.,1.9951695175

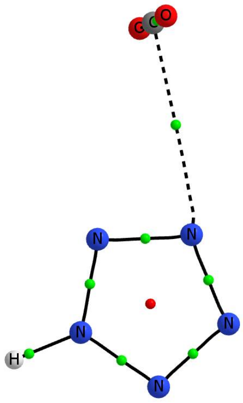	1H-pent-23p<br>MP2= -462.16229813 NIMAG= 0<br>C,2.432388604,0.0245513847,0.<br>O,2.441912584,0.0206615398,1.1701009336<br>O,2.441912584,0.0206615398,-1.1701009336<br>N,-0.588378221,-0.7291173407,0.<br>N,-0.4977457489,0.5884504839,0.<br>N,-1.8877778532,-0.9524049398,0.<br>N,-1.7278046183,1.1181518216,0.<br>N,-2.620270706,0.1453970592,0.<br>H,-2.2873570246,-1.8816424785,0.

Table S4. Structures (Å), total energies (a.u.), and molecular graphs of hydrogen-bonded CO_2:azole complexes

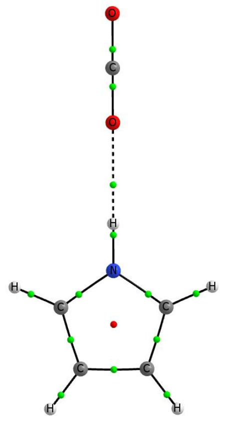	1H-pyrr<br>MP2= -398.07162177 NIMAG= 0<br>C,0.,0.,-3.4964986496<br>O,0.,0.,-2.3246952146<br>O,0.,0.,-4.6638813805<br>N,0.,0.,0.8506168761<br>C,0.,1.121913066,1.63440219<br>C,0.,-1.121913066,1.63440219<br>C,0.,0.7082353985,2.95473723<br>C,0.,-0.7082353985,2.95473723<br>H,0.,0.,-0.1554371991<br>H,0.,2.1061835051,1.1984878319<br>H,0.,-2.1061835051,1.1984878319<br>H,0.,-1.3577931483,3.8138016119<br>H,0.,1.3577931483,3.8138016119
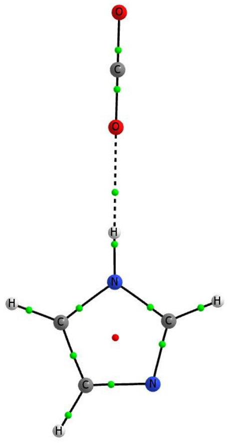	1H-imid<br>MP2= -414.11793162 NIMAG= 0<br>C,-2.2880865408,1.0527201927,0.<br>O,-1.2045314969,0.6055206279,0.<br>O,-3.3664833789,1.4982647037,0.<br>N,1.6814714667,-0.6608037946,0.<br>C,1.9566471959,-1.9941108613,0.<br>N,3.2602597584,-2.2219588731,0.<br>C,3.840497088,-0.9777297706,0.<br>C,2.8781525027,0.008008757,0.<br>H,0.7650991289,-0.2425547706,0.<br>H,2.935646823,1.0820017335,0.<br>H,1.1837254828,-2.7439990447,0.<br>H,4.9102364902,-0.8555450598,0.

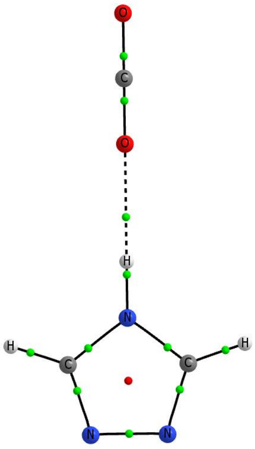	4H-124tri<br>MP2= -430.13999255 NIMAG= 0<br>C,0.,0.,-3.3356438<br>O,0.,0.,-2.1629184<br>O,0.,0.,-4.50172987<br>N,0.,0.68967067,3.03863268<br>N,0.,-0.68967067,3.03863268<br>C,0.,1.07845138,1.77737114<br>C,0.,-1.07845138,1.77737114<br>N,0.,0.,0.94929401<br>H,0.,0.,-0.05871838<br>H,0.,2.09817587,1.43406185<br>H,0.,-2.09817587,1.43406185
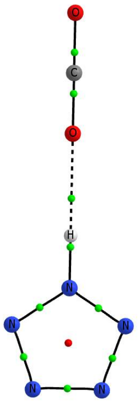	1H-pent<br>MP2= -462.16473811 NIMAG= 0<br>C,0.,0.,-3.2133506138<br>O,0.,0.,-2.0403326781<br>O,0.,0.,-4.3782790519<br>N,0.,0.,0.9478427127<br>N,0.,1.1050176622,1.6682706473<br>N,0.,0.6698525848,2.9154421267<br>N,0.,-0.6698525848,2.9154421267<br>N,0.,-1.1050176622,1.6682706473<br>H,0.,0.,-0.0664301855
